# Associations of Proanthocyanidin Intake with Renal Function and Clinical Outcomes in Elderly Women

**DOI:** 10.1371/journal.pone.0071166

**Published:** 2013-08-05

**Authors:** Kerry L. Ivey, Joshua R. Lewis, Wai H. Lim, Ee M. Lim, Jonathan M. Hodgson, Richard L. Prince

**Affiliations:** 1 School of Medicine and Pharmacology Sir Charles Gairdner Hospital Unit, University of Western Australia, Perth, Western Australia, Australia; 2 Department of Endocrinology and Diabetes, Sir Charles Gairdner Hospital, Perth, Western Australia, Australia; 3 Department of Renal Medicine, Sir Charles Gairdner Hospital, Perth, Western Australia, Australia; 4 School of Medicine and Pharmacology, Royal Perth Hospital, University of Western Australia, Perth, Western Australia, Australia; The University of Manchester, United Kingdom

## Abstract

**Background:**

Progression to chronic renal failure involves accelerated atherosclerosis and vascular calcification. Oxidative stress and endothelial dysfunction play a role in renal failure pathophysiology. In addition to improving vascular health and function, proanthocyanidins have been shown to exert renoprotective effects in animal models. Thus we hypothesize that proanthocyanidins may contribute to the maintenance of healthy renal function.

**Objective:**

Determine the association of habitual proanthocyanidin intake with renal function and the risk of clinical renal outcomes in a population of elderly women.

**Design:**

948 women aged over 75 y, free of prevalent renal disease at baseline, were randomly selected from ambulant Caucasian women. Proanthocyanidin consumption was determined using a validated food frequency questionnaire and the United States Department of Agriculture proanthocyanidin food content database. Fasting serum cystatin C and creatinine were assessed at baseline. Renal failure hospitalisations and deaths were assessed over 5 years of follow-up through the Western Australia Data Linkage System.

**Results:**

Compared to participants with low consumption, participants in the highest tertile of proanthocyanidin intake had a 9% lower cystatin C concentration (P<0.001). High proanthocyanidin consumers were at 50% lower risk of moderate chronic kidney insufficiency, and 65% lower risk of experiencing a 5-year renal disease event (P<0.05). These relationships remained significant following adjustment for renal disease risk factors and diet-related potential confounders.

**Conclusion:**

Increased consumption of proanthocyanidins was associated with better renal function and substantially reduced renal associated events, which has been supported by mechanistic and animal model data. Proanthocyanidin intake should be further examined as a dietary contributor to better renal health.

## Introduction

Chronic kidney disease (CKD) represents a growing public health issue [1]. The pathophysiology of CKD involves several mechanisms that are analogous to cardiovascular disease [2]. Oxidative stress, atherogenesis, nitric oxide homeostasis, and endothelial function play important roles in the pathogenesis of these diseases [3]–[7]. Ageing is associated with structural and functional changes in the kidneys [8], resulting in impaired renal function [9]. Reduced glomerular filtration rate (GFR) is a risk factor for atherosclerotic vascular disease [10], [11].

A recent meta-analysis has shown that when compared to creatinine, serum cystatin C may be a more accurate measurement of GFR [12], especially in the elderly [13]. Cystatin C provides early indications of renal dysfunction [14] and is less affected by muscle mass, weight, height, age and sex [15]. Recognised risk factors for elevated cystatin C levels include traditional atherosclerotic vascular disease risk factors [16], [17]. However, the effect of dietary constituents on cystatin C levels remains uncertain.

Proanthocyanidins are a diverse group of plant-derived oligomeric compounds, and are members of the flavonoid group of molecules. There is mounting evidence that proanthocyanidins and proanthocyanidin-rich foods and beverages contribute to vascular health and reduce risk of vascular outcomes [18], [19] through acting as free radical scavengers, reducing platelet aggregation and blood pressure, and improving nitric oxide homeostasis and endothelial function [20]–[24]. Although these vascular benefits of flavonoids are not limited to the proanthocyanidin class of flavonoids, based on mounting mechanistic and animal model data showing improved renal function and outcomes with proanthocyanidin supplementation [25]–[29], it appears the renoprotective benefit is limited to the specific flavonoid class of proanthocyanidins. As such, we hypothesise that via similar mechanisms, proanthocyanidins may contribute to maintenance of healthy renal function and slows GFR decline over time. Therefore this study aimed to explore the association of habitual intake of proanthocyanidins with renal function and the risk of CKD and renal failure events in a population of elderly women.

## Subjects and Methods

### Participants

Following completion of a 5-year prospective, randomized, controlled trial of oral calcium supplements to prevent osteoporotic fractures [30], the women were then invited to take part in a follow-up study beginning in 2003: the Calcium Intake Fracture Outcome Age Related Extension Study. At baseline (2003), the women were older than 75 y, and a total of 948 had complete proanthocyanidin, cystatin C and renal outcome data, and did not have prevalent renal disease at baseline. The Human Research Ethics Committee of the University of Western Australia approved the study, and written informed consents were obtained from all participants.

### Renal function

Serum was collected after an overnight fast, and serum cystatin C was quantified using a fully automated particle-enhanced immunoturbidimetric assay with Sentinel Diagnostics reagents (Sentinel CH, Milan, Italy) on the Architect ci 16200 System (Abbott Laboratories, Illinois, USA) according to the manufacturer instructions; (intra-assay CV<1.5%, inter-assay CV<1%). Baseline serum creatinine was assessed in 918 participants and was analysed in 2005 using an isotope dilution mass spectrometry traceable Jaffe kinetic assay for creatinine on a Hitachi 917 analyzer (Roche Diagnostics GmbH, Mannheim Germany).

### Estimated glomerular filtration rate

In order to evaluate renal function, the estimated GFR (eGFR) was calculated in mL/min/1.73 m^2^ from serum creatinine and cystatin C using the methods recently published in the New England Journal of Medicine [31]. The CKD-EPI creatinine-cystatin C equations for participants with a serum cystatin C (Scys)≤0.8 mg/L were: serum creatinine (Scr)≤0.7 mg/dl, eGFR = 130×(scr/0.7)^−0.248^×(Scys/0.8)^−0.375^×0.995^Age^ or Scr>0.7 mg/dl, eGFR = 130×(scr/0.7)^−0.601^×(Scys/0.8)^−0.375^×0.995^Age^. Estimated GFR for participants with a Scys>0.8 mg/L were calculated using the following equations: Scr≤0.7 mg/dl, eGFR = 130×(scr/0.7)^−0.248^×(Scys/0.8)^−0.711^×0.995^Age^ or Scr>0.7 mg/dl, eGFR = 130×(scr/0.7)^−0.601^×(Scys/0.8)^−0.711^×0.995^Age^. Using this equation, moderate chronic kidney insufficiency was defined as eGFR<60 mL/min/1.73 m^2^.

### Renal disease events

5-year incidence of acute or chronic renal failure events causing hospitalization or death was retrieved from the Western Australian Data Linkage System (WADLS) for each of the study participants from baseline. WADLS provides a complete validated record of every participant's primary diagnosis hospitalizations and cause of death, if applicable, from the coded records of the death certificate. Renal failure events were defined using primary and additional diagnosis codes from the International Classification of Diseases, Injuries and Causes of Death Clinical Modification (ICD-9-CM) [32] and the International Statistical Classification of Diseases and Related Health Problems, 10^th^ Revision, Australian Modification (ICD-10- AM) [33]. These codes included; glomerular diseases (ICD-9-CM codes 580–583, ICD-10- AM codes N00-08); renal tubulo-interstitial diseases (ICD-9-CM codes 593.3–593.5, 593.7 and 590–591, ICD-10- AM codes N09-16); renal failure (ICD-9-CM codes 584–586, ICD-10- AM codes N17-19); and hypertensive renal disease (ICD-9-CM code 403, ICD-10- AM codes I12). The search for renal failure death ICD codes included all available diagnostic information that comprised Parts 1 and 2 of the death certificate and the principal diagnosis in the inpatient data. All diagnosis text fields from the death certificate were used to ascertain the cause(s) of deaths where these data were not yet available from the WADLS.

### Baseline chronic kidney disease risk assessment

Baseline medical histories were obtained from all participants and were coded using the International Classification of Primary Care – Plus method [34], as previously described in Ivey et al. [35]. Previous atherosclerotic vascular disease was determined using verified hospitalisations from 1980–2003 from the Western Australian Data Linkage System. Participants maintained on anti-hypertensive medications at baseline were considered to have prevalent hypertension.

Smoking status was coded as non-smoker or ex-smoker/current smoker if they had smoked more than 1 cigarette per day for more than 3 months at any time in their life. Baseline weight was assessed using digital scales with participants wearing light clothes and no shoes. Baseline height was assessed using a stadiometer, and the body mass index (BMI) was calculated in kg/m^2^.

### Dietary assessment

Baseline dietary intake was assessed by a validated semi-quantitative food frequency questionnaire (FFQ) developed by the Anti-Cancer Council of Victoria [36]. Energy and nutrient intakes were estimated based on frequency of consumption and an overall estimate of usual portion size [37]. A beverage intake questionnaire [38] which quantified habitual beverage consumption during the preceding year, aws also completed at baseline. Specifically participants reported average daily tea and coffee consumption over the past 12 months.

### Proanthocyanidin intake

Estimates of the proanthocyanidin content of foods in the FFQ and beverage questionnaire were derived from the Proanthocyanidin food content database [39]. The method of computing proanthocyanidin content of foods was similar to that outlined in Sesso et al. [40]. Specifically, for each food, we computed the sum of assessed proanthocyanidins by summing the proanthocyanidin dimers, trimers, 4–6 mers, 7–10 mers and polymers. When multiple varieties of a food listed in the FFQ database were reported, the average proanthocyanidin content of all similar varieties was computed, consistent with the descriptors used in the FFQ output. Foods in the FFQ that were not in the USDA proanthocyanidin database were assumed to contain no proanthocyanidins. Intake of proanthocyanidins in mg/d was calculated by multiplying the estimated intake (g edible portion/d) from FFQ and beverage questionnaire, with the proanthocyanidin class content (mg/g edible portion) of each food item on the questionnaires.

A similar method was adopted to calculate an estimate of the intake of non-proanthocyanidin flavonoids. The Flavonoid 2.1 and Isoflavone 2.0 [41] food content databases were used to determine flavonoid content of food items, by summing mg/g edible portion for each of the individual compounds in the flavonol, flavan-3-ol, flavone, flavanone, anthocyanidin and isoflavone flavonoid classes present in each of the databases.

### Statistics

Before commencing statistical analysis, a pre-specified analytical protocol was produced. The relationship between proanthocyanidin intake and baseline cystatin C concentration was examined in regression analysis using unadjusted and multivariate-adjusted models. This included continuous variables energy and protein intake, BMI and age, and dichotomous variables antihypertensive use, prevalent cardiovascular disease (CVD), diabetes and history of smoking. The multivariate analysis included 932 participants due to missing data for one or more of the atherosclerotic vascular disease risk factors.

Participants were then divided into 3 groups based on tertiles of proanthocyanidin intake for further analysis by analysis of variance (ANOVA). Renal disease event odds ratio (OR) and 95% confidence intervals (CI) were obtained using binary logistic regression of flavonoid intake by standard deviation (SD) scores, and multivariable ANCOVA of tertiles of proanthocyanidin consumption.

Post hoc comparisons were only made after the main effect of the factor was found to be significant in the multivariable analyses. Stepwise linear regression of proanthocyanidin intake and cystatin C and Stepwise logistic regression of flavonoid class intake and renal disease events were used to account for potential covariance of independent variables. The multivariable candidate variables included antihypertensive use, BMI, prevalent CVD and diabetes, history of smoking, age, and intakes of energy, non-proanthocyanidin flavonoids, protein, fluid, phosphate, calcium, carbohydrate, and saturated fat at baseline. P≤0.05 was the level of significance used to determine which multivariate candidate variables were included in the final model. The data were analysed using SPSS (version 15; SPSS Inc, Chicago, IL) and SAS (Version 9, SAS Institute Inc., Chicago, IL).

## Results

Mean total proanthocyanidin intake was 215±147 mg/d, range 18–1728 mg/d. Over 50% of total proanthocyanidin intake came from fruit (89±63 mg/d), chocolate (43±75 mg/d), and alcoholic beverages (32±86 mg/d).

The baseline characteristics of the cohort are shown in **Table 1**. Mean cystatin C concentration at baseline was 1.18 (±0.29) mg/L, and over the 5-year follow-up period, 60 (6%) of participants experienced a renal disease event.

**Table 1:** Baseline, lifestyle and cardiovascular risk factors by tertiles of proanthocyanidin intake.

	Low intake	Moderate intake	High intake
	<141 mg/d	141–<229 mg/d	≥229 mg/d
Number of subjects	316 (33)	316 (33)	316 (33)
**Renal disease risk factors**			
Age (years)	80±3	80±3	80±3
History of smoking [n (%)]	78 (25)	82 (26)	95 (30)
Previous ASVD [n (%)]^a^^,^ b	65 (21)	40 (13)	49 (16)
Previous diabetes [n (%)]	22 (7)	15 (5)	16 (5)
Antihypertensive medication use [n (%)]	183 (58)	160 (51)	173 (55)
Body mass index (kg/m^2^)	27±5	27±5	27±4
Energy intake (kJ/d)^a^	5612±1604	6730±1867	8262±3145
Protein (g/d)^a^	64±22	77±28	93±41
**Dietary intake**			
Non-proanthocyanidin flavonoids (mg/d)^a^	371±222	508±246	553±261
Fluid (mL/d)^a^	2416±793	2680±750	2734±887
Phosphate (mg/d)^a^	1178±359	1413±439	1655±612
Calcium (mg/d)^a^	812±290	917±307	997±344
Sodium (mg/d)^a^	1703±559	2015±704	2348±1045
Saturated fat (g/d)^a^	21±10	24±11	30±16
Carbohydrate (g/d)^a^	149±43	180±47	217±80

Results are mean ± SD or n (%) where appropriate. (n = 948).

a represents significantly different (P<0.05) by ANOVA or chi-squared test where appropriate.

b ASVD: atherosclerotic vascular disease.

### Renal function by serum cystatin C

The concentration of cystatin C was inversely associated with intake of proanthocyanidins; unadjusted standardised ß = −0.086, P = 0.008. This association remained significant in the fully adjusted model which included age, antihypertensive use, energy and protein intake, body mass index, prevalent atherosclerotic vascular disease and diabetes and smoking history; standardized ß = −0.134, P<0.001.

This relationship was explored further by dividing the population into tertiles of proanthocyanidin consumption (**Table 2**). In unadjusted and multivariate-adjusted models, participants in the highest tertile of proanthocyanidin intake had a lower cystatin C concentration than those in the lowest tertile; 7% and 9% reduction respectively. A similar trend was obtained when using serum creatinine concentration as an indicator of renal function; multivariate adjusted standardized ß = −0.073 and P = 0.052.

**Table 2:** Baseline cystatin C concentration according to groups of proanthocyanidin intake.

	Low intake	Moderate intake	High intake	P value
	<141 mg/d	141–<229 mg/d	≥229 mg/d	
Number of subjects	316 (33)	316 (33)	316 (33)	
**Cystatin C** (mmol/L)				
Unadjusted^1^	1.23±0.02^a^	1.17±0.02^b^	1.14±0.02^b^	**0.001**
Age-adjusted^2^	1.23±0.02^a^	1.17±0.02^b^	1.14±0.02^b^	**<0.001**
Multivariate-adjusted model^2^	1.24±0.02^a^	1.18±0.02^b^	1.13±0.02^c^	**<0.001**

1 Results are mean ± SEM.

2 Results are least-squared mean ± SEM by ANCOVA.

a,b,c represents significantly different by LSD (P<0.05).

Multivariate-adjusted model: antihypertensive use, energy and protein intake, BMI, prevalent CVD and diabetes, history of smoking and age.

### Renal function by eGFR using the CKD-EPI equation (creatinine and cystatin C)

The mean eGFR of participants was 61.7±13.7 ml/min/1.73 m^2^, and 367 (39%) participants had moderate chronic kidney insufficiency at baseline, as defined by an eGFR<60 ml/min/1.73 m^2^. In unadjusted and fully adjusted models, the risk [multivariate adjusted OR (95% CI) per SD] of CKD was significantly associated with intake of proanthocyanidins; 0.76 (0.63–0.91), P = 0.003. Participants in the highest tertile of proanthocyanidin intake had a 50% lower risk of having moderate chronic kidney insufficiency than those in the lowest tertile (**Table 3**).

**Table 3:** Relationship between proanthocyanidin intake and 5-year hospitalisation or death renal failure events.

	Low intake	Moderate intake	High intake	P value
	<141 mg/d	141–<229 mg/d	≥229 mg/d	
Number of subjects	316 (33)	316 (33)	316 (33)	
**eGFR<60 ml/min/1.73 m^2^** [n(%)]	151 (49)	117 (38)	99 (33)	
Unadjusted	1.00 (referent)	0.64 (0.47–0.89)*	0.50 (0.36–0.70)*	**<0.001**
Age-adjusted	1.00 (referent)	0.63 (0.45–0.87)*	0.48 (0.34–0.67)*	**<0.001**
Multivariate-adjusted	1.00 (referent)	0.61 (0.43–0.87)*	0.44 (0.30–0.65)*	**<0.001**
**Renal failure events** [n(%)]	32 (10)	16 (5)	12 (4)	
Unadjusted	1.00 (referent)	0.47 (0.25–0.88)*	0.35 (0.18–0.69)*	**0.004**
Age-adjusted	1.00 (referent)	0.47 (0.25–0.88)*	0.35 (0.17–0.68)*	**0.003**
Multivariate-adjusted	1.00 (referent)	0.53 (0.27–1.05)	0.40 (0.18–0.89)*	**0.048**

Results are OR (95% CI) by logistic regression.

* represents significantly different from referent (P<0.05).

Multivariate-adjusted model: antihypertensive use, energy and protein intake, BMI, prevalent CVD and diabetes, history of smoking and age.

### Chronic kidney disease and clinical outcomes

Compared to the lowest tertile, participants in the highest tertile of proanthocyanidin intake were at 65% lower risk of experiencing a 5-year renal disease event (**Table 3**).

Five-year renal disease hospitalisation incidence was significantly different across proanthocyanidin consumption tertiles by chi-squared test (P = 0.015). In the lowest tertile of proanthocyanidin consumption, there were 28 (9%) renal hospitalisations, compared to 15 (5%) and 12 (4%) in the moderate and high proanthocyanidin consumption groups, respectively. Although not significant (P = 0.087), a similar trend was observed with renal disease associated mortality. There were 9 renal failure deaths (3%) over the 5 year follow up in the lowest proanthocyanidin consumers, whereas in the moderate and high consumption groups, there were 3 (1%) and 3 (1%) deaths, respectively. The lack of significant association with renal disease mortality is likely due to lack of power to detect the association.

### Potential dietary confounders

To account for additional diet related potential confounders, a stepwise linear regression model of cystatin C concentration that included proanthocyanidin intake, and baseline renal disease risk factors and dietary intake variables outlined in Table 1 was performed. The most parsimonious model consisted of: proanthocyanidin intake, body mass index, age, anti-hypertensive medication use, previous atherosclerotic vascular disease and intakes of non-proanthocyanidin flavonoids, saturated fat and total fluid intake.

Similarly, the addition of proanthocyanidin intake significantly improved the logistic regression model predictions for renal outcomes. In stepwise logistic regression for risk of chronic kidney disease, the most parsimonious model included proanthocyanidin intake SD score, age, antihypertensive medication use, body mass index, and previous atherosclerotic vascular disease. In stepwise logistic regression for renal event, the most parsimonious model was proanthocyanidin intake SD score, age, and histories of both atherosclerotic vascular disease and diabetes.

### Non-proanthocyanidin flavonoids

A similar approach was used to identify the relationship between intake of other flavonoids and renal outcomes. Despite being significantly associated with cystatin C concentration (multivariate adjusted standardised ß = −0.105, P = 0.001), intake of non-proanthocyanidin flavonoids was not significantly associated with risk of moderate chronic kidney insufficiency or renal event; multivariate adjusted OR per SD = 0.899 (P = 0.163) and multivariate adjusted OR per SD 0.783 (P = 0.109), respectively.

## Discussion

This study of elderly women is the first prospective study to suggest a potential role of proanthocyanidins in maintaining renal function and preventing renal disease events. Total proanthocyanidin intake was beneficially associated with cystatin C concentration. Proanthocyanidin consumption was also inversely associated with risk of moderate chronic kidney insufficiency and renal failure event in this cohort.

Total proanthocyanidin consumption of ≥141 mg/d was associated with a significantly better cystatin C concentration. This relationship was sufficiently robust to remain after adjustment for identified baseline and dietary risk factors. Compared to subjects with low proanthocyanidin intake, those with high consumption had a 7% lower cystatin C concentration. This difference in cystatin C concentration is likely to be of clinical significance as a 0.18 mg/L lower cystatin C concentration has been associated with a 33% lower risk of mortality [42].

The clinical significance of this relationship is further supported by our findings that participants with habitual high proanthocyanidin consumption had lower risks of moderate chronic kidney insufficiency and renal failure events. These results are reinforced by recent meta-analyses showing that moderate intake of wine [43] and chocolate [44], both rich sources of proanthocyanidins [39], are associated with reduced risk of cardiovascular disease; an independent risk factor for impaired kidney function and renal disease [45], [46]. The ability of this relationship to extend to public health outcomes has been demonstrated by results of a controlled trial showing that when compared to a protein restricted diet, a polyphenol rich diet, low in carbohydrates and iron, was 40–50% more effective at reducing risk of renal events [47].

Our results showing that proanthocyanidins may contribute to renal health are supported by results of *in vitro* studies, studies using animal models, and randomised controlled trials in humans investigating potential mechanisms and pathways [21], [25], [29], [48]–[55]. There is now direct evidence that proanthocyanidins specifically can improve renal health in animal models by reducing oxidative stress, improving antioxidant defence potential, and reducing oxidative renal injury [25], [29], [48]. Animal models have also shown that proanthocyanidins and proanthocyanidin rich foods improve renal function and reduce apoptosis of tubular and interstitial cells [49], [50].

Another possible mechanism for renal protection by proanthocyanidins is by augmenting nitric oxide status and improving endothelial function [21], [51]–[53]. More direct evidence that proanthocyanidins may be responsible for improved endothelial function derives from trials using flavonoid-rich cocoa [54] and grape seed extract [55]. It is important to note that proanthocyanidins with more than 2 flavonoid units are not absorbed intact. Prior to absorption, proanthocyanidins are metabolised to phenolic acids by gut bacteria [56]. As such, it is likely that phenolic acid compounds are responsible for any physiological effects of proanthocyanidin consumption [57].

The level of flavonoid intake varies greatly across geographical areas [58], and to our knowledge, this is the first summary of proanthocyanidin intake in elderly Australian women. The mean proanthocyanidin intake reported in this study is greater than that reported for women from other Western countries [59], [60]. However, this may be due to relatively greater flavonoid intake in Australia [61].

The paper has the following limitations. Firstly as an epidemiological paper the outcomes are based on post hoc classifications of patients and thus does not allow true randomisation. Secondly the metrics used for assessment of proanthocyanidin consumption may not truly reflect consumption of this cohort as the technique uses analytical assays of US foods which may differ from Australian food items, and as such, the regional variation of proanthocyanidin content of foods has not been accounted for in this investigation. However the analytical data on intake were calculated before examination of the relation to clinical outcome data and was subject to rigorous covariate analysis in an attempt to identify important co-correlates that may have accounted for the observed relations. Identification of causality is further limited by the complexity of proanthocyanidin compounds and the variability of the proanthocyanidin content of foods. However, the strength of the association is such that despite these factors, the association remains significant even after adjustment for baseline, dietary and lifestyle risk factors.

To our knowledge, this is the first study to investigate the relationship between proanthocyanidin intake and renal outcomes in humans. In this cohort of elderly women, proanthocyanidin intake was associated with improved renal function and reduced risk of CKD and renal disease associated events. The renoprotective benefits of proanthocyanidins appear to be independent of traditional risk factors and dietary variables known to affect renal health, suggesting a habitual diet high in proanthocyanidins may play a role in preventing renal function decline and renal diseases. In addition to being of clinical significance, it is likely these findings will be of public health relevance, as 141 mg/d proanthocyanidin consumption is equivalent to an approximate daily intake of 50 g beans, 60 g nuts or 20 g chocolate. Ultimately, in order to make public health recommendations regarding proanthocyanidin intake, further observational and intervention trials are necessary to establish the clinical benefits on renal health.
